# First case of fungal keratitis due to *Aspergillus minisclerotigenes* in Iran

**DOI:** 10.18502/cmm.5.2.1162

**Published:** 2019-06

**Authors:** Mahmoud Karimizadeh Esfahani, Alireza Eslampoor, Somayeh Dolatabadi, Mohammad J. Najafzadeh, Jos Houbraken

**Affiliations:** 1 Department of Parasitology and Mycology, Faculty of Medicine, Mashhad University of Medical Sciences, Mashhad, Iran; 2 Eye Research Centre, Faculty of Medicine, Mashhad University of Medical Sciences, Mashhad, Iran; 3 Faculty of Engineering, Sabzevar University of New Technology, Sabzevar, Iran; 4 Westerdijk Fungal Biodiversity Institute, Utrecht, the Netherlands

**Keywords:** Aspergillus minisclerotigenes, Fungal keratitis, Iran

## Abstract

**Background and Purpose:**

Herein, we report the first case of fungal keratitis due to *Aspergillus* *minisclerotigenes* in a 68-year-old rural woman admitted to the Ophthalmology Center of Khatam-Al-Anbia Hospital in Mashhad, northeast of Iran.

**Case report:**

The patient presented with severe pain, burning, foreign body sensation, and reduced vision in her right eye. She had long-term uncontrolled diabetes and was not able to close her eye due to an anatomical problem with the eyelid. The cornea smear sample was cultured, and the fungus was initially identified as *Aspergillus* *flavus*. The isolated strain was further identified by sequencing a part of the calmodulin gene as *A. minisclerotigenes*. The patient did not respond to any antifungal treatments (e.g., amphotericin B and voriconazole drops, and fluconazole 300 mg/day); therefore, she was eventually subjected to corneal transplantation surgery.

**Conclusion:**

Fungal keratitis can be caused by the less common species. The reliable identification of the causative agents can be accomplished by the implementation of molecular methods.

## Introduction

Mycotic keratitis, also known as fungal keratitis, is a fungal infection of the cornea due to a defect in the corneal epithelium presenting with the inflammation of the corneal stroma [1]. Fungal keratitis can cause visual loss and accounts for up to 44% of all cases of microbial keratitis, depending on the geographical location [2]. According to the World Health Organization, 1.5-2 million individuals annually go blind due to keratitis [3]. 

Keratitis is the most important cause of ocular morbidity and mainly occurs in outdoor and agricultural workers as an occupational disease [4, 5]. Moreover, this disease is mostly common in the tropical and subtropical areas. Fungal keratitis may affect individuals in any age group and gender; however, the males performing agricultural or other outdoor work are the most susceptible group [6]. 

The local predisposing factors for fungal keratitis include trauma (with plant material, animal origin, and dust particles), contact lenses, and iatrogenic agents (following cataract surgery, refractive surgery, and penetrating keratoplasty). In addition, the systemic predisposing factors for this disease are diabetes mellitus, rheumatoid arthritis, human immunodeficiency virus infection, and the use of topical corticosteroids or traditional eye medicines [7, 8].

The common causative agents of cornea infection include species belonging to the genera *Fusarium*, *Aspergillus*, *Curvularia*, *Bipolaris*, and *Candida *[9]. *Aspergillus* species has been frequently reported as the etiological agent of fungal keratitis in tropical countries, such as India [10]. The most common species are *A. flavus*, *A.* *fumigatus*, *A. terreus*, and *A. niger,* while *A. glaucus*, *A. ochraceus*, *A. tamari*, *A. brasiliensis*, *A. tubingensis*, and *A.* *viridinutans* are less frequently occurring [10, 11]. 

It is crucial to identify the causative agents of keratomycosis at the species level as these agents show differences in their pathogenicity and intrinsic antifungal susceptibility. Herein, we reported the first case of fungal keratitis caused by *Aspergillus* *minisclerotigenes*, a species belonging to section *Flavi* of the genus *Aspergillus*.

## Case report

The case was a 68-year-old female from Bardaskan, Northeast of Iran, admitted to the Ophthalmology Center of Khatam-Al-Anbia Hospital in Mashhad, Iran. The patient was a farmer dealing with livestock. She presented with severe pain, burning, foreign body sensation, and reduced vision in her right eye. She had long-term uncontrolled diabetes and was unable to close her right eye due to the anatomical problem of the eyelid. The patient did not remember if she had received any inoculation in the past.

**Figure 1:**
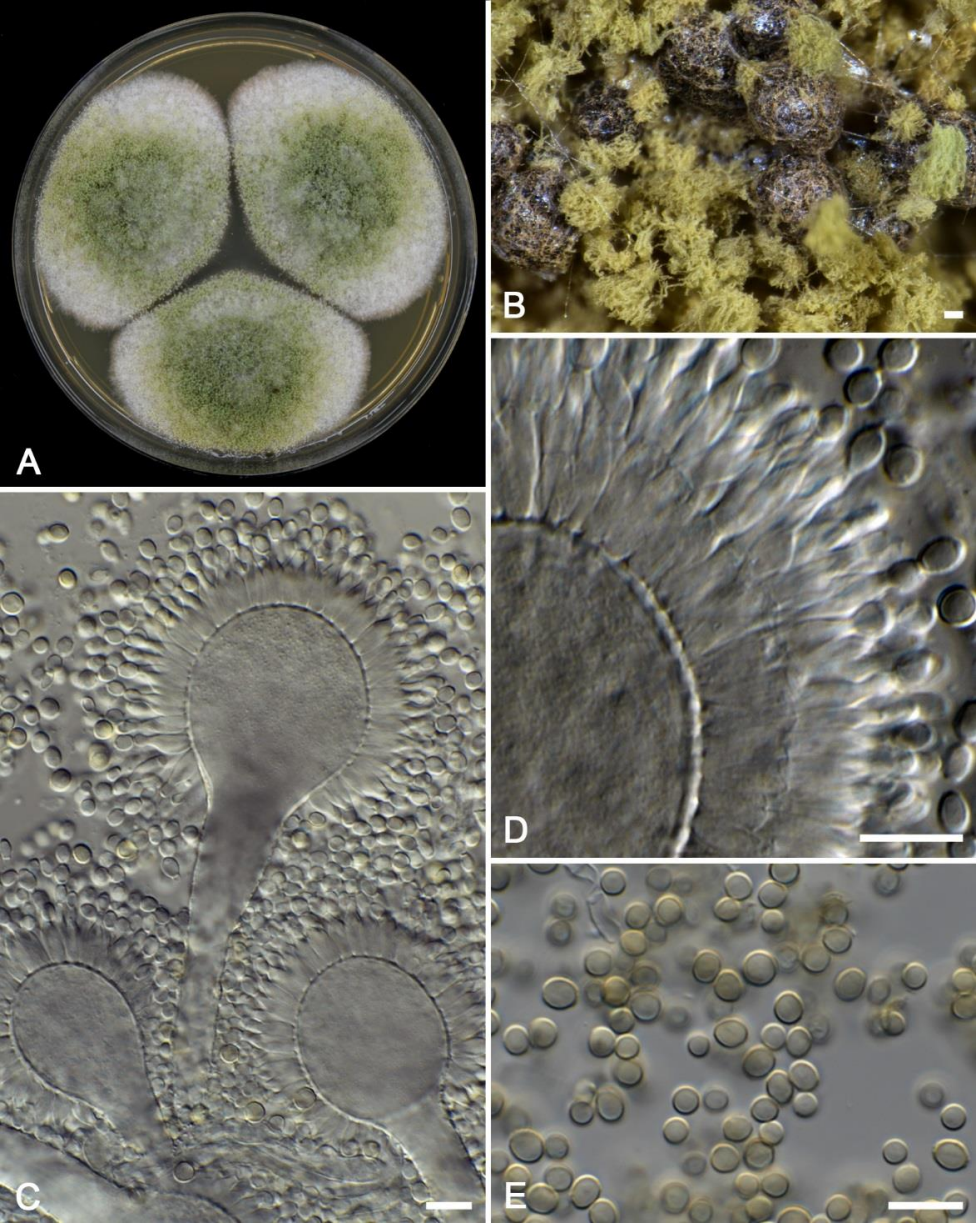
*Aspergillus minisclerotiogenes* CBS 145094; A) a 7-day-old colony on MEA, B) details of colony showing black colored sclerotia, C) uniseriate conidiophores, D) details of a biseriate conidial head, E) conidia (Scale bar=10 µm)

Direct microscopic analysis of the corneal scraping smear showed branched septated mycelium indicating a fungal infection. The corneal scraping samples were cultured on malt extract agar and incubated at 25°C and 37°C for 7 days. Both uni- and biseriate *Aspergillus* heads and black colored sclerotia resembling *A. flavus *were observed and the latter measured 300-500 µm in diameter (Figure 1). 

**Figure 2:**
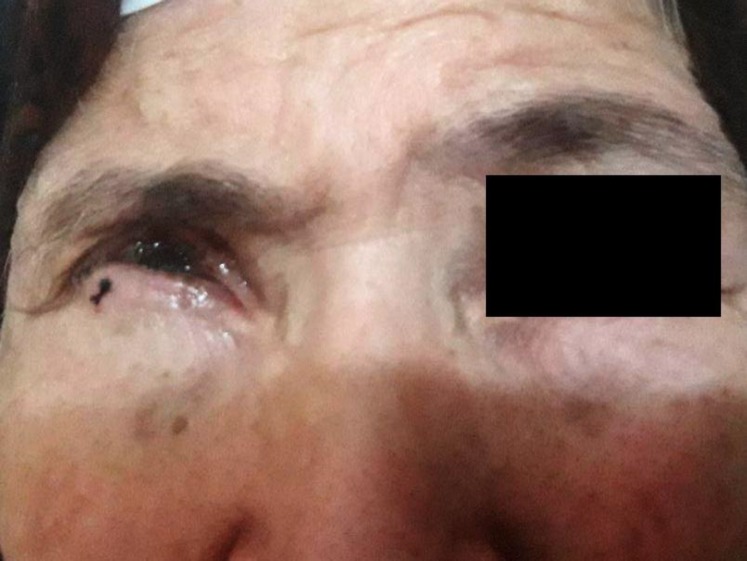
A photo from the eye of the patient two weeks after corneal transplant surgery

The patient was initially misdiagnosed; accordingly, she did not respond to any of the antifungal therapies (e.g., amphotericin B (1 mg/ml, Q2H) and voriconazole (1 mg/mL, Q2H) eye drops, and fluconazole [300 mg/day]). This irresponsiveness may be due to the delay in the treatment and spread of infection, which eventually led to a corneal transplantation surgery after obtaining informed consent (Figure 2). 

Final identification was performed using partial calmodulin (*CaM*) gene sequencing. DNA extraction was accomplished by means of the Utraclean^TM^ Microbial DNA isolation kit (MoBio, Solana Beach USA). Amplification of a part of the *CaM* gene and sequencing were performed using two primer pairs, namely cmd5 (CCGAGTACAAGGAGGCCTTC) and cmd6 (CCGATAGAGGTCATAACGTGG) [12], following the study by Frisvad et al. [13]. 

The partial *CaM* sequences of our isolate were 100% identical to those of *A. minisclerotigenes* deposited in the GenBank under the accession numbers of HM803026, HM803016, and HM803014. The generated sequence in this study was deposited in the GenBank under accession number MG490650. In addition, the isolate was deposited in the culture collection of the Westerdijk Fungal Biodiversity Centre, Utrecht, the Netherlands, under collection number CBS145094.

## Discussion

*Aspergillus* species are ubiquitous and common outdoor fungi. The pre dominant presence of these fungi in the environment exposes the outdoor workers and farmers to contamination mostly occurring through the penetration of thorns and wood chips. However, fungal keratitis can also occur after such operations as laser in situ keratomileusis, and cataract [14] or glaucoma surgery [15]. The first reported case of keratitis due to *Aspergillus* species was reported in a farmer who got struck in the eye through an oat chaff [16]. 

*Aspergillus* species are the common cause of keratomycosis in tropical and subtropical countries. *Aspergillus* *flavus* has been reported as the main agent of fungal keratitis. The identification of *Aspergillus* species based on morphological characters is difficult, or even impossible, due to the existence of cryptic species. This variation can be reflected later on with regard to the response of these species to different antifungals. Therefore, a reliable identification of these etiological agents could be achieved by molecular methods.

Our isolated strain was able to produce sclerotia on CYA media although the size was larger as commonly observed in *A. minisclerotigenes* which was first described as “*A. flavus* Group II” by Geiser et al. [17]. The occurrence of *A. minisclerotigenes* might have been overlooked (and probably reported as *A. flavus*) as many species reported in older literature were only identified on the basis of their macroscopic and microscopic characteristics. 

## Conclusion

The present study was the first report of fungal keratitis due to *A. minisclerotigenes* in the world. Fungal keratitis can be caused by the less common species. The reliable identification of the causative agents can be accomplished by the implementation of molecular methods.
